# When moments matter: Finding answers with rapid exome sequencing

**DOI:** 10.1002/mgg3.1027

**Published:** 2019-12-24

**Authors:** Zöe Powis, Kelly D. Farwell Hagman, Kirsten Blanco, Margaret Au, John M. Graham, Kathryn Singh, Natalie Gallant, Linda M. Randolph, Meghan Towne, Jesse Hunter, Deepali N. Shinde, Erika Palmaer, Brian Schoenfeld, Sha Tang

**Affiliations:** ^1^ Ambry Genetics Aliso Viejo CA USA; ^2^ Department of Pediatrics Cedars Sinai Medical Center Los Angeles CA USA; ^3^ Memorial Care Health System Genetics Clinic Long Beach CA USA; ^4^ Division of Medical Genetics Children's Hospital Los Angeles Los Angeles CA USA

**Keywords:** clinical utility, exome, genetic testing, rapid exome

## Abstract

**Background:**

When time is of the essence in critical care cases, a fast molecular diagnosis is often necessary to help health care providers quickly determine best next steps for treatments, prognosis, and counseling of their patients. In this paper, we present the diagnostic rates and improved quality of life for patients undergoing clinical rapid exome sequencing.

**Methods:**

The clinical histories and results of 41 patients undergoing rapid exome sequencing were retrospectively reviewed.

**Results:**

Clinical rapid exome sequencing identified a definitive diagnosis in 13/41 (31.7%) and other relevant findings in 17 of the patients (41.5%). The average time to verbal report was 7 days; to written report was 11 days.

**Conclusions:**

Our observations demonstrate the utility and effectiveness of rapid family‐based diagnostic exome sequencing in improving patients care.

## Graphical abstract

The ability of a health care provider to order testing and counsel families within 7 days is advantageous for patients. About 31.7% received a diagnosis and 92.7% of providers reported ordering testing to aid in prognosis and medical management decisions, including potential end of life decisions.

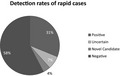

## 1 INTRODUCTION

Exome sequencing is a powerful technology that diagnoses roughly 30% of patients with a broad spectrum of unknown genetic conditions, a rate that is two to three times that of traditional genetic testing methods (Farwell Hagman et al., 2016; Farwell et al., 2014; Iglesias et al., 2014; Lee et al., 2014; Yang et al., 2014), at about half to one‐quarter of the cost (Monroe et al., 2016; Soden et al., 2014). Initially, health care providers were discouraged from using exome sequencing in certain situations as it did not “support short turnaround times” (ACMG Board of Directors, 2012). Since its clinical availability in 2011, the advancement of exome technology now brings more rapid diagnoses. A faster turnaround time (TAT) impacts the clinical utility of exome sequencing as highlighted in one study showing that results were deemed clinically beneficial in 32.6% of diagnosed patients with standard TAT (median 136 days), and 65% in patients with rapid exome sequencing (median 12–23 days). Stark et al. also demonstrated cost savings per diagnosis and in treatments after diagnosis in individuals undergoing rapid exome sequencing compared to those with standard TATs (Stark et al., 2018).

Specifically, a shortened diagnostic journey in neonates has been shown to aid the process for appropriate, targeted treatments, accurate prognosis, and counseling (Willig et al., 2015). These first studies investigated only a small number of patients in selected populations (such as prenatal or neonatal) undergoing rapid exome (Chandler et al., 2018; Willig et al., 2015). Herein, we present detailed clinical characteristics of an unselected group of individuals undergoing rapid exome testing to determine a diagnostic rate and investigate its utility. We also include case studies to illustrate how exome sequencing may aid in diagnosis and treatment.

## 2 MATERIALS AND METHODS

### 2.1 Ethical compliance

Solutions Institutional Review Board determined the study to be exempt from the Office for Human Research Protections Regulations for the Protection of Human Subjects (45 CFR 46) under category 4.

### 2.2 Subjects

A total of 41 patients were ascertained sequentially through clinical samples sent to Ambry Genetics for rapid exome sequencing. The initial rapid TAT time was 2–5 weeks in 2014 (14 patients) and reduced to a verbal report within 8 days (with a full written report including Sanger confirmation within 14 days) in 2016 (27 patients). Patients were counseled and consented for testing by the ordering provider and/or other health care provider. Parental and/or additional informative family member samples (when available) were also sent for these patients and utilized for variant phasing and analysis and co‐segregation analysis.

### 2.3 Testing methods

#### 2.3.1 Diagnostic exome sequencing

All patients' clinical and testing histories, along with pedigrees provided by referring physicians, were reviewed and summarized by a team of board certified genetic counselors with previous clinical experience. DNA isolation, exome library preparation, sequencing, bioinformatics, and data analysis were performed as previously described (Farwell Hagman et al., 2016; Farwell et al., 2014). Screening of established mitochondrial mutations was also included in the analysis. Genes were classified as characterized (known to cause Mendelian disease) or uncharacterized (analysis of genes not previously associated with disease) based on Ambry's clinical validity assessment criteria (Smith et al., 2017).

Careful review of Integrative Genomics Viewer (IGV) was performed so that verbal results could be given before Sanger confirmation. All relevant alterations were confirmed by Sanger sequencing.

#### 2.3.2 Secondary findings

Secondary or incidental findings (SF) as recommended by ACMG were reported when ordered (Kalia et al., 2017).

#### 2.3.3 Statistical analysis

Statistical analysis was performed using the Fisher's exact test.

## 3 RESULTS

### 3.1 Characteristics of patients

A total of 41 patients were identified for evaluation. Demographic information including primary reasons for referral is summarized in Table 1. 51.2% of patients tested were female. Additionally, 51.2% of patients tested were less than 1 year of age, with additional 41.5% also pediatric patients over age 1 year. 46.3% of patients tested had a reported abnormal brain MRI, 36.6% of patients had hypotonia, 36.6% of patient had reported seizures/epilepsy, and 36.6% of patients had reported intellectual disability and/or developmental delay. 75.6% of patients tested had neurologic findings, and 41.5% had musculoskeletal/structural anomalies.

**Table 1:** Demographic information

Characteristic	Overall (*n* = 41)	Positive (*n* = 13)
Gender		
Male	20 (48.8%)	10 (76.9%)^a^
Female	21 (51.2%)	3 (23.1%)
Ethnicity		
African American	2 (4.9%)	1 (7.7%)
Asian	8 (19.5%)	4 (30.8%)
Caucasian	19 (46.3%)	6 (46.2%)
Hispanic	1 (2.4%)	0 (0.0%)
Mixed ethnicity	5 (12.2%)	1 (7.7%)
Unknown	6 (14.6%)	1 (7.7%)
Age at testing		
<1 year	21 (51.2%)	8 (29.4%)
1–17 years	17 (41.5%)	5 (29.4%)
Adults	3 (7.3%)	0 (0%)
Clinical history		
Multiple congenital anomalies	7 (17.1%)	2 (15.4%)
MR/ID/DD	15 (36.6%)	7 (53.8%)
ASD	3 (7.3%)	1 (7.7%)
Psychiatric	3 (7.3%)	1 (7.7%)
Seizures/epilepsy	15 (36.6%)	3 (23.1%)
Movement disorders	12 (29.3%)	4 (30.8%)
Brain MRI positive	19 (46.3%)	6 (46.2%)
Phenotype is progressive	10 (24.4%)	3 (23.1%)
Dysmorphic features	13 (31.7%)	4 (30.8%)
FTT/undergrowth	9 (22.0%)	4 (30.8%)
Overgrowth	0 (0.0%)	0 (0.0%)
Hypotonia	15 (36.6%)	6 (46.2%)
Organ system involvement		
Allergy/immunologic/infectious	10 (24.4%)	4 (30.8%)
Audiologic/otolaryngologic	2 (4.9%)	1 (7.7%)
Cardiovascular	11 (26.8%)	3 (23.1%)
Craniofacial	6 (14.6%)	2 (15.4%)
Dental	0 (0.0%)	0 (0.0%)
Hematologic	9 (22.0%)	3 (23.1%)
Dermatologic	1 (2.4%)	0 (0.0%)
Endocrine	4 (9.8%)	0 (0.0%)
Gastrointestinal	11 (26.8%)	3 (23.1%)
Genitourinary	6 (14.6%)	2 (15.4%)
Metabolic/biochemical	8 (19.5%)	2 (15.4%)
Musculoskeletal/structural	17 (41.5%)	5 (38.5%)
Neurologic	31 (75.6%)	10 (76.9%)
Obstetric	0 (0.0%)	0 (0.0%)
Oncologic	1 (2.4%)	0 (0.0%)
Ophthalmologic	5 (12.2%)	1 (7.7%)
Pulmonary	10 (24.4%)	6 (46.2%)^a^
Renal	6 (14.6%)	1 (7.7%)

a Statistically significant.

Sixteen patients (39.0%) did not have uncharacterized genes analyzed, due to lack of an informative trio for exome sequencing, clinician order (opted out of novel gene analysis), or a positive finding found in a characterized gene. The average time to verbal result disclosure (when available) was 7 days (range 5–10 days), and for Sanger confirmation and a written report was 11 days (range 7–15 days). The results did not change from verbal to written report for any case. The 27 patients who had exome sequencing with an 8‐day TAT were less likely to have had previous genetic testing. None of these previously ordered genetic tests were reported as diagnostic. In total, 12 (44.4%) patients had no previous testing, and 15 (55.5%) patients had one or more previous genetic test. Of the 14 patients who received testing with a 2–5 weeks TAT, only two (14.2%) had no previous genetic tests and 12 (85.7%) had one or more previous genetic test. Patients with positive results were more likely to have had other molecular genetic testing (*p* = .0017) and less likely to have had only one genetic test performed (*p* = .0326).

Overall, 19 (46.3%) had a chromosome microarray, eight (19.5%) had a karyotype, six (14.6%) had a gene panel, five (12.2%) had a single gene test, two (4.9%) had previous mitochondrial testing, and five (12.2%) had other molecular genetic testing. Fifteen patients (36.6%) had no previous postnatal genetic testing reported; 12 of these (80%) were patients who had exome sequencing performed within the shortened TAT. Health care providers reported ordering testing with the intent to assist with management decisions in 23 (59.0%) patients. Fifteen (36.6%) patients were reported as critically ill, two (4.9%) postmortem, and one (2.4%) patient having testing traveled from a foreign country and wished to have results before returning.

### 3.2 Patients with relevant findings

Clinical diagnostic exome sequencing identified potentially relevant findings in 17 of the patients (41.5%), including 31.7% (13/41) of which were in characterized genes (Table S1). One patient had novel candidate gene findings and three patients had uncertain findings. There were no likely positive findings. None of the cases has yet had reclassified results. There were no statically significant findings in detection rate by age group.

Of the positive findings, 7/13 (53.8%) were in autosomal dominant genes, 4/13 (30.8%) were autosomal recessive, and 2/13 (15.4%) were X‐linked recessive. About 8/13 (61.5%) cases were inherited and the remainder (38.5%) were confirmed to be de novo through parental testing.

Although 48.8% of patients were males, they were likely to have a positive finding (*p* = .0203). About 2.4% of patients were reported with pulmonary findings and this group were more likely to have positive results (*p* = .0485) (Table 1). Primary reasons for referral for testing were mixed and there were no statistically significant differences in the reasons for referral (Table 2).

**Table 2:** Primary indication for testing

Primary indication for referral	Number of probands (*n* = 41)	Positive/likely positive (*n* = 13)
Two or more major or three or more minor malformations	5 (12.2%)	2 (15.4%)
Cancer susceptibility	1 (2.4%)	0 (0.0%)
Cardiovascular	1 (2.4%)	0 (0.0%)
Gastrointestinal	1 (2.4%)	0 (0.0%)
Hypotonia	2 (4.9%)	1 (7.7%)
Immune	4 (9.8%)	2 (15.4%)
Metabolic	5 (12.2%)	1 (7.7%)
Neurodevelopmental NOS	8 (19.5%)	2 (15.4%)
Neuromuscular NOS	3 (7.3%)	2 (15.4%)
Neuropathy	1 (2.4%)	0 (0.0%)
Other movement disorder	2 (4.9%)	0 (0.0%)
Seizures	4 (9.8%)	1 (7.7%)
Undergrowth/FTT	1 (2.4%)	0 (0.0%)

NOS, not otherwise specified.

### 3.3 Secondary findings

Of the 41 patients, nine (22.0%) did not have/declined SF testing. All patients undergoing SF testing had negative results.

### 3.4 Case report one

This male proband was born at 39 1/7 weeks to a 35‐year‐old Caucasian woman and her 38‐year‐old Asian partner, weighing 3,635 g (71.73rd centile), with length 50.8 cm (65.79th centile), and occipital frontal circumference (OFC) 34.5 cm (51.23rd centile) via repeat cesarean section following in vitro fertilization (IVF. An older son is healthy, family history was noncontributory, and consanguinity was denied. Prenatal ultrasound demonstrated left hydronephrosis, left foot postaxial polydactyly, and polyhydramnios, and after birth, a patent foramen ovale and pelviectasis with a duplicated left ureter were noted. Prenatal chromosome microarray and karyotype were normal. Newborn screening, including cystic fibrosis, was normal. He was admitted to the neonatal intensive care unit (NICU) at day 1 of life due to presumed bowel obstruction. At 1 week of age, he had an intestinal resection due to severe bowel obstruction with meconium ileus. Hirschsprung's disease was ruled out by biopsy. Two weeks after the initial resection, he continued to have absent bowel function and a second resection was performed. Thick, sticky meconium was noted at the anastomotic site, and normal bowel function returned thereafter, allowing him to be discharged home at age 24 days.

Rapid trio exome sequencing and testing for cystic fibrosis sequencing (including deletion/duplication analysis of *CFTR*) were received at the lab for testing concurrently at day 17. At day 22 of age, the genetic counselor was called and given the verbal report of two biparentally inherited alterations of uncertain significance in *GUCY2C* (c.2575A > G [p.I859V] and c.2864_2865delCCinsTA [p.S955L]), and *CFTR* was 100% covered at 10x on exome. *CFTR* sequencing and deletion/duplication analysis was reported as normal at day 23.

GUCY2C interacts with CFTR and encodes the guanylate cyclase C (GC‐C) receptor, an epithelial transmembrane receptor expressed throughout the intestine (OMIM_601330). GC‐C plays a role in the regulation of electrolyte and fluid release to the intestinal lumen, as well as epithelial differentiation, proliferation, and tumorigenesis (von Volkmann et al., 2017). Autosomal recessive alterations in this gene have been associated with meconium ileus (OMIM_614665).

The c.2575A > G (p.I859V) alteration was observed in 92 of 269,026 total alleles studied (0.03%), having been seen in 0.5% (91/18,174) East Asian alleles and <0.001% (1/124,596) European non‐Finnish alleles and conserved throughout vertebrates (Lek et al., 2016). The p.I859V alteration is also predicted to be probably damaging by Polyphen and deleterious by SIFT in silico analyses (Adzhubei et al., 2010; Kumar, Henikoff, & Ng, 2009).

Identification of the *GUCY2C* alterations led to a definitive diagnosis and accurate genetic counseling that alleviated possible concerns regarding cystic fibrosis. Further evaluations and possible treatment for CF (sweat chloride test, referral to CF clinic, pancreatic enzymes) were deemed unnecessary and discharge planning was altered accordingly. As results were available prior to the patient's discharge, the family received in‐person genetic counseling the same day the results were provided by the lab instead of waiting weeks or months for an outpatient appointment.

### 3.5 Case report two

The second proband is of mixed ethnic background (Italian, German, Ashkenazi Jewish, French Canadian, and Mexican). She is a 3‐month‐old female born at 41 4/7 weeks by normal spontaneous vaginal delivery (NSVD) (birthweight 2.95 kg, length 46 cm, OFC 33 cm) to a 21‐year‐old G1 P0‐1 woman. Amniotic bands were reported but did not wrap around the fetus. Mom denied all teratogens. Fetal movement and amniotic fluid volume were normal. Maternal multiple marker screening was negative. The pregnancy history was significant for a 30 lb. weight loss in the first trimester. The baby was discharged from the hospital at 1 day of age. The proband was initially taken to emergency room at 2 months due to left lower leg edema and fussiness. She was afebrile and vital signs were initially normal but she later became cyanotic and unresponsive, requiring resuscitation. Apneic episodes occurred both during sleep and wakefulness 1–3 times per day and self‐resolving after approximately 2 min associated with cyanosis and altered mental status with normal EEGs. She was also noted to be lethargic, and pale with poor feeding.

Genetic evaluation at age 3 months noted microcephaly is proportionate to overall habitus, shallow orbits causing prominent appearance of eyes (maternal trait), large eyes, anteverted nares, nasal tip extending past alae, and high arched palate. At that time, she kept her hands fisted with cortical thumbs although she did open her hands spontaneously, weak cry, weak suck, facial and oral strength appeared weak, diffusely hypotonic with poor head control, diffusely weak, consistently fixated, and did not reach or grasp. She had a frenulectomy and feeding tube placed at 4 months.

The family history is negative for similarly affected individuals but otherwise significant for a strong history of cancer in maternal relatives. Past testing history included normal DEB breakage, chromosome analysis, single nucleotide polymorphism (SNP) microarray (hg19), Prader‐Willi/Angelman methylation, lysosomal enzyme screen, 7‐dehydrocholesterol, creatine kinase, sweat test, and metabolic testing. A brain MRI showed decreased white matter and a slightly thin corpus callosum. An echocardiogram, EKG, EEG, chest x‐ray, and skeletal survey were normal.

Rapid exome sequencing showed a pathogenic de novo mutation in *ASXL3* c.1389_1390delTG (p.C463*). Pathogenic alterations in *ASXL3* are associated with autosomal dominant Bainbridge–Ropers syndrome (BRPS) (OMIM_615485). BRPS is characterized by global developmental delay, hypotonia, moderate to severe intellectual disability with severe speech delay, feeding difficulties during infancy with poor appetite and/or vomiting, failure to thrive, and characteristic facial dysmorphism (Bainbridge et al., 2013; Balasubramanian et al., 2017; Dinwiddie et al., 2013; Hori et al., 2016). Typical dysmorphic features found in patients with BRPS (many seen in the patient) include long face, high and prominent forehead with metopic prominence, down‐slanting palpebral fissures, with metopic prominence, sparse eyebrows, bulbous nose, strabismus, high narrow palate, prominent columella, small nasal alae, anteverted nares, and downturned corners of the mouth (Kuechler et al., 2017). Seizures, hyperventilation, and skeletal features have been reported in a few patients (Balasubramanian et al., 2017; Kuechler et al., 2017). Our patient had periodic breathing and apneic spells. Her length, weight, and head circumference were below the third percentile.

Identification of the *ASXL3* alterations led to an end of an extensive diagnostic journey with numerous previous tests within the first 3 months of life. She was evaluated by pulmonary, gastroenterology, medical genetics, and rehabilitation medicine, and diagnosed with central and obstructive sleep apnea, dysphagia, and probable esotropia, pending ophthalmology evaluation. She was receiving 1‐L oxygen by nasal cannula 24 hr per day as of 4 months of age.

## 4 DISCUSSION

The clinical utility of rapid diagnostic exome is unmatched. Rapid exome sequencing delivers additional value beyond traditional measures in making individualized medical management decisions and determining accurate prognosis much earlier than with traditional genetic testing. Initial concerns over the time to obtain results have been eliminated with the use of rapid exome as it is now as fast as or faster than even single gene testing. The ability of a health care provider to order testing and counsel families within 7 days is advantageous for patients, especially as 31.7% received a diagnosis and 92.7% of providers reported ordering testing to aid in prognosis and medical management decisions, including potential end of life decisions (including those with a negative result). The fact that the number of declined secondary findings is higher than previously reported may indicate that patients are critically ill and/or primary results are of integral importance to the patients.

Whereas exome can be cost‐effective and rapid test can provide more timely results; the nature of rapid exome makes it more expensive than customary exome. Although it can be more expensive, these benefits outweigh the costs for some patients. Providers may want to consider rapid exome on a case by case basis for their patients.

Previous studies have only investigated rapid testing in critically ill neonates or studied infants without specifically studying rapid exome (Meng et al., 2017; Willig et al., 2015). While this is the first study to study an unselected group of patients receiving rapid exome, the majority were pediatric patients. Some pediatric patients are especially suited for rapid exome as the results in our study and previous have shown significant effects on clinical decision‐making (Meng et al., 2017; Willig et al., 2015).

One provider reported ordering testing for a patient traveling in for evaluation. Such patients can benefit from treatment, recurrence risk counseling, and follow‐up. While these cases may not be common, it demonstrates another circumstance for utility of rapid exome testing.

Interestingly, positive results were more likely to be found in male patients, which were not explained by inheritance as the majority of findings were autosomal. Those with pulmonary findings were also more likely to be positive perhaps related to the high number of neonatal patients with pulmonary findings. Despite this, age had no impact on diagnostic yield; therefore, rapid exome sequencing can be beneficial for all,

Patients with positive results were less likely to have had multiple prior genetic tests. It is possible a more complex diagnostic journey incidates a more complex phenotype that is difficult to diagnose with exisiting molecular technologies. Potentially health care providers might order exome as a first‐tier test for these patients, giving a timelier and cost‐effective result.

In summary, our data suggest that when assessing patients with a suspected genetic condition, clinical rapid exome sequencing may be superior to traditional genetic testing when a rapid diagnosis is expedient due to patient condition. The option for rapid testing with results in 7 days gives the medical provider information that can potentially improve outcomes for a vulnerable population. Previous studies have focused on critically ill neonates undergoing rapid exome testing (Willig et al., 2015). This work suggests that appropriate use of clinical rapid exome sequencing may augment the rate of a cost‐effective genetic diagnosis for a variety of patients.

## CONFLICT OF INTEREST

Zöe Powis, Kelly D. Farwell Hagman, Kirsten Blanco, Meghan Towne, Jesse Hunter, Deepali N. Shinde, Erika Palmaer, Brian Schoenfeld, and Sha Tang are employed by Ambry Genetics. Exome sequencing is among the commercially available tests. All other authors have nothing to declare.

## AUTHOR CONTRIBUTIONS

ZP, KFH, and ST had the original idea for the study. KB compiled the study database. KFH and ST critically reviewed the study design, contributed to the collection and analysis of the data and the results. ZP checked and analyzed the data, computed the results, and wrote the initial draft of the manuscript including the text, tables, and figures. MA, JMG, KH, NG, LMR provided patient data critical to the results. KFH and ZP worked together on statistical analyses. ZP, KFH, MA, JMG, KH, NG, LMR, MT, JH, DNS, EP, BS, and ST all critically reviewed and commented on and approved the final manuscript.
