# Supplementary material for: When moments matter: Finding answers with rapid exome sequencing
Source: Mol Genet Genomic Med. 2019 Dec 24;8(2):e1027. doi: 10.1002/mgg3.1027 (PMC7005623; doi:10.1002/mgg3.1027)
Supplement: Supplementary file 1 [file MGG3-8-e1027-s001.docx]

| **Gene** | **Associated Condition** |
| --- | --- |
| *ACTA1* | ACTA1-related congenital myopathy (OMIM_161800) |
| *ACTC1* | Atrial septal defect 5 (OMIM_ 612794) |
| *ASXL3* | Bainbridge-Ropers syndrome (BRPS) (OMIM_615485) |
| *ATP1A2* | Alternating hemiplegia of childhood (OMIM_104290) |
| *CACNA1C* | Timothy syndrome (OMIM_601005) |
| *CLN8* | Neuronal ceroid lipofuscinosis 8 (NCL8) (OMIM_600143) |
| *FANCA* | Fanconi anemia, complementation group A (OMIM_227650) |
| *G6PD* | G6PD Deficiency (OMIM_305900) |
| *IL2RG* | Severe combined immunodeficiency, X-linked (OMIM_300400) |
| *MAGEL2* | Prader-Willi-like syndrome (OMIM_615547) |
| *POLG* | POLG-related disorder (OMIM_174763) |
| *SPAST* | Spastic paraplegia 4, autosomal dominant (OMIM_182601) |
| *SPTBN2* | Spinocerebellar ataxia, autosomal recessive 14 (OMIM_615386) |
| *SOX10* | SOX10-related peripheral demyelinating neuropathy, central dysmyelination, Waardenburg syndrome with or without Hirschsprung disease (PCW/PCWH syndrome) (OMIM_ 602229) |
